# Face-to-Face Meetings with Neurosurgical Patients Before Hospital Discharge: Impact on Telephone Outreach, Emergency Department Visits, and Hospital Readmissions

**DOI:** 10.1089/pop.2019.0038

**Published:** 2020-03-11

**Authors:** Franz H. Vergara, Jean E. Davis, Chakra Budhathoki, Nancy J. Sullivan, Daniel J. Sheridan

**Affiliations:** ^1^ Bloomberg School of Public Health, The Johns Hopkins University, Baltimore, Maryland.; ^2^ Goldfarb School of Nursing at Barnes-Jewish College, St. Louis, Missouri.; ^3^ School of Nursing, The Johns Hopkins University, Baltimore, Maryland.; ^4^ Texas A&M University, College of Nursing, Bryan, Texas.

**Keywords:** telephone follow-up, reach rate, readmissions, emergency visits, face-to-face meetings

## Abstract

The Johns Hopkins Community Health Partnerships (JCHiP) was developed in 2010 within the Johns Hopkins Health Systems. As part of JCHiP, the Patient Access Line call center was created. The average telephone reach rate at The Johns Hopkins Hospital in 2014 was only 53%. In a population of adult neurosurgical patients, this study aimed to: determine the impact of face-to-face meetings with neurosurgical patients before hospital discharge on telephone follow-up (TFU) reach rates, and determine the association between TFU reach rates and subsequent emergency department (ED) visits and hospital readmission rates. This quasi-experimental study used a posttest-only research design with a comparison group. Two adult inpatient neurosurgical units at the Johns Hopkins Hospital were selected as the intervention and comparison groups. A convenience sampling technique was used. Face-to-face meetings pre hospital discharge resulted in a TFU reach rate of 97.7% on the intervention unit while the comparison unit had only a 76.1% TFU reach rate (*P* < .001). Reached patients had fewer ED visits (7.8%) than not reached patients (17.4%); however, the difference was not statistically significant (*P* = .138). Reached patients also had fewer hospital readmissions (3.3%) than not reached patients (8.7%); this also was not statistically significant (*P* = .214). This study demonstrated that face-to-face meetings with neurosurgical patients prior to discharge increased TFU rates. Results were statistically significant. ED visits and hospital readmissions were also reduced in reached patients and the findings were clinically significant.

## Introduction

**FIG. 1.**
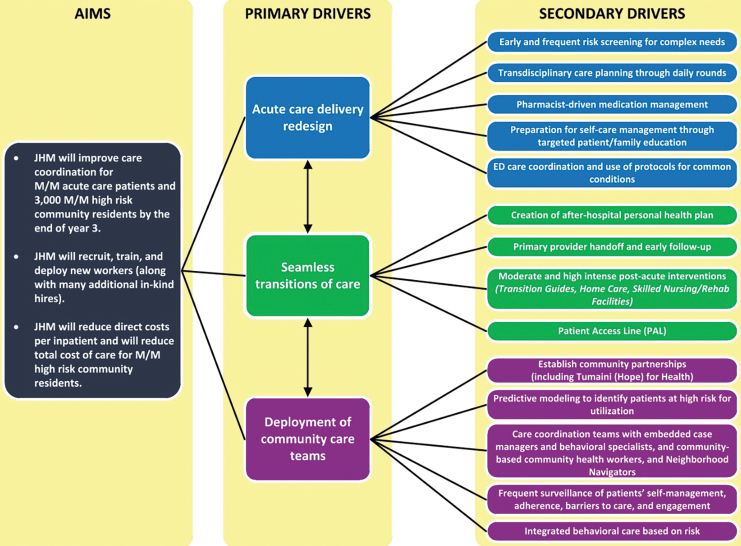
JCHiP driver diagram for care coordination. ED, emergency department; JHM, Johns Hopkins Medicine; M/M, Medicare/Medicaid.

In 2010, the Centers for Medicare & Medicaid Services awarded the Johns Hopkins Health Systems a grant to create and design a comprehensive and integrated health care program, the Johns Hopkins Community Health Partnerships (JCHiP). JCHiP was a complementary bundle of interventions with primary and secondary drivers (Figure 1) designed to improve access to care for high-risk adults and reduce hospital readmissions in East Baltimore, MD.^1^ When deployed, it was associated with lower spending and improved health care outcomes by reducing an aggregate total cost of care of $59.8 million for Medicare and Medicaid participants.^2^

As a part of JCHiP, the Patient Access Line (PAL) call center was created to serve as a secondary driver to promote seamless transitions of care from hospital to home.^3^ Hsiao et al^4^ published a detailed explanation of the implementation of JCHiP and the PAL call center in a recent study. Initially, the PAL call center was staffed by 5 telephonic registered nurse case managers with the primary goal of conducting telephone follow-up (TFU) post hospital discharge. The average TFU reach rate at Johns Hopkins Hospital in 2014 was 53%.^5^ There were very few published studies on improving telephone outreach. Most studies focused on medical patients.^6,7^ Rates of telephone outreach were unknown for neurosurgical patients. Also, there were few recent studies linking TFU to subsequent emergency department (ED) visits and 30-day hospital readmissions.^3,4,8^ Hoyer et al emphasized that “patients at highest risk for rehospitalization were also the least likely to receive the assigned care coordination intervention,”^8 (p 626)^ such as the PAL call. Therefore, it is imperative to develop methods to engage the hardest to reach patients to fully deploy the various drivers of the JCHiP program such as TFU.

The aims of this study were to:

1. Determine the impact of a pre hospital discharge face-to-face meeting on the post hospital TFU telephone reach rate of neurosurgical patients; and
2. Determine the association of successfully reaching patients post hospital discharge with subsequent ED visits and readmission rates of patients admitted to an adult neurosurgical patients unit.

## Methods

### Study design and setting

A prospective posttest-only quasi-experimental research design with a comparison group was used to address the study aims. To examine causality, a quasi-experimental design was selected, as true randomization was not feasible during the study time period and randomly allocating participants to intervention and comparison groups was not practical.^9^ A convenience sampling technique was employed as a measure to recruit as many participants as possible to increase the power of the study.

The study was conducted on 2 neurosurgical units (named Unit A and Unit B for study purposes) servicing similar populations at the Johns Hopkins Hospital. After flipping a coin, Unit A was selected as the intervention ward, with Unit B serving as the comparison.

### Intervention

#### Face-to-face meeting intervention

The study used a 1-time, face-to-face meeting intervention conducted by the corresponding author that lasted approximately 6–10 minutes for each participant. Face-to face meeting as an intervention was guided by the elements of the Transitions Theory.^10^ At each face-to-face meeting intervention the investigator:

- introduced himself and greeted the patient by shaking hands;
- asked the patient's permission to sit down so that the investigator could speak without looking down at the patient;
- informed the patient of the purpose of TFU post hospital discharge; and
- assisted the patient with completing a patient handout that would be used by the investigator to contact the patient post hospital discharge.

The patient handout requested the following information:

- the best phone number(s) to reach the patient;
- the best time and date for TFU; and
- a reminder of paperwork and items needed at the time of the phone call.

Each patient signed the patient handout and took this document with her/him upon discharge. This both demonstrated agreement about the scheduled TFU and served as an appointment card for the TFU. The photocopy of the signed patient handout was de-identified, and a unique identification code was assigned.

Twenty-four to 72 hours after hospital discharge, a PAL call was conducted for all eligible patients based on the date and time agreed to during the face-to-face meeting. If participants were not reached at the agreed-upon time for the telephone call, 2 more attempts were made to reach the patient telephonically. After 3 telephone call attempts, if the patient did not answer or was unavailable, attempts to conduct TFU were discontinued.

#### Routine care

Patients admitted to the comparison group received care as usual, which included up to 3 TFU call attempts made by telephonic nurse case managers without meeting patients prior to hospital discharge. The traditional workflow process and task list for routine care included the following:

- Creating daily unit-based discharge reports from Research Electronic Data Capture (REDCap);
- Conducting post hospital discharge telephone calls using a prescribed script;
- Contacting the discharging medical providers, home care services, or pharmacists as indicated;
- Answering questions about self-care management needs; and
- Documenting the TFU in the electronic medical record (EMR) at the end of the telephone call.

Intervention and comparison groups both received the routine care of TFU post hospital discharge. However, the comparison group did not receive a face-to-face meeting prior to hospital discharge and TFU.

### Study sample, screening, and enrollment

The intervention and comparison units had a similar mix of patients and used a novel care coordination program from JCHiP, with TFU being a component of discharge planning. Eligible patients in this research study met the following criteria:

- postoperative patients on the neurosurgical wards;
- low-to-moderate risk of hospital readmission;
- ages 18 years or older; and
- speak and understand English.

Patients with the following post hospital discharge needs and conditions were excluded:

- need for skilled nursing home care or transition guide services;
- planned transfer to a skilled nursing facility, rehabilitation unit, or assisted living facility;
- readmitted patients from another nursing unit or hospital;
- case management by Johns Hopkins International (JHI) staff;
- prior referral to hospice or palliative care agencies;
- left hospital against medical advice; and
- lacking capacity to consent.

#### Enrollment

**FIG. 2.**
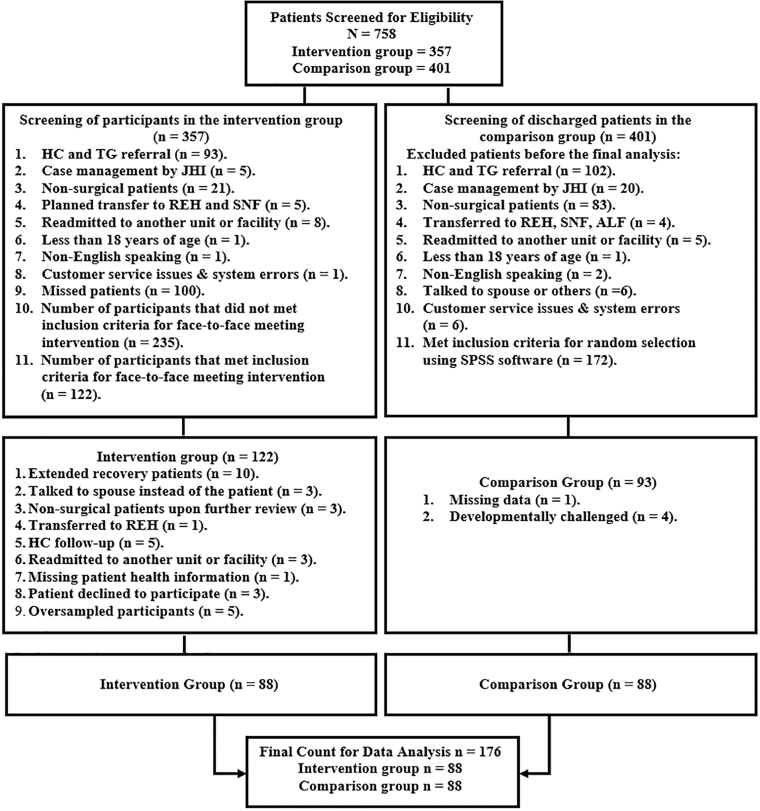
CONSORT flow diagram for screening eligible patients. ALF, assisted living facility; HC, home care; JHI, Johns Hopkins International; REH, rehabilitation unit; SNF, skilled nursing facility; TG, transition guide.

Face-to-face meeting interventions were conducted at the intervention unit from June 13, 2016, to September 16, 2016. The number of face-to-face meeting interventions per week varied depending on the number of eligible patients for each day of data collection. The Consolidated Standards of Reporting Trials (CONSORT) flow diagram of Kuriyama et al^11^ was used to elucidate the screening strategies for eligible participants (Figure 2). A total of 758 patients were screened as possible research participants. After using the random selection functionality of IBM SPSS (IBM Corporation, Armonk, NY)^12^ the intervention and comparison groups were each allocated 88 subjects.

While conducting face-to-face meetings with the intervention group, a retrospective chart review was conducted to determine the independence of research participants because of the probability of hospital readmission of patients in the comparison group. The review prevented enrolling research participants twice between the intervention and comparison groups. Therefore, the subjects in each group remained mutually exclusive.

### Statistical analysis

A power analysis was conducted a priori to ensure sufficient sample size to achieve adequate power.^9^ Sample size estimates using medium-range effect sizes were calculated using the Power Analysis and Sample Size online computer software based on Cohen's^13^ formula. A medium effect size of 0.30 using a 1 degree-of-freedom chi-square test with a significance level of 0.05 was selected to obtain a power of 80%. Based on the estimated medium effect size as calculated, at least 88 participants were needed in each group (intervention and comparison units), for a total minimum of 176 patients. Nonparametric statistical methods (Pearson chi-square test and Fisher exact tests) were employed to determine any differences between categorical sociodemographic and surgical variables. Nominal data were analyzed as to whether the patient was reached or not reached (yes or no). Pearson chi-square test and cross tabulations were used to compare proportions of reached or not reached patients, patients who had an ED visit or not, and patients readmitted or not.^14^

### Data collection

Data were collected and managed using REDCap, a secure, web-based software application for collecting research information.^15^ REDCap is the internal documentation system of the PAL department. In addition, a retrospective EMR review (using EPIC; Epic Systems Corporation, Verona, WI) was conducted 30 days after hospital discharge for each research participant from the intervention and comparison units. The EMR review determined:

- how many patients were either reached or not reached by TFU;
- how many phone call attempts were made to reach the patients;
- how many and which patients visited or did not visit the ED within 30 days of hospital discharge; and
- how many and which patients were readmitted or not readmitted 30 days after hospital discharge.

### Ethical consideration and data security

An Institutional Review Board application was submitted to Johns Hopkins Medicine and this study was classified as a quality improvement and deemed exempt for full review because of its less than minimal risk to participants. Data were de-identified through substitution by creating a reidentification table.^16^ The folders containing the reidentification table and master keys table were saved on the firewalled and protected server of the Johns Hopkins Medical Institutions.

## Results

Of the 15 sociodemographic and background variables, 4 demonstrated a significant difference between the intervention and comparison groups. Employment status (*P* = .014), educational attainment (*P* = .002), having children younger than age 18 years living at home (*P* = .010), and hospital service (*P* = .039) were significantly different between the intervention and comparison groups (Table 1).

**Table 1.** Sociodemographic Variables of Participants and Differences Between Groups (N = 176)

Characteristics	Intervention (n = 88)		Comparison (n = 88)		Total		
	n	%	n	%	N	%	*P*
Age in Years							0.939^a^
18–29	9	10.2	12	13.6	21	11.9	
30–39	13	14.8	11	12.5	24	13.6	
40–49	22	25.0	17	19.3	39	22.2	
50–59	20	22.7	23	26.1	43	24.4	
60–69	17	19.3	16	18.2	33	18.8	
70–79	5	5.7	7	8.0	12	6.8	
≥80	2	2.3	2	2.3	4	2.3	
Sex							1.000^b^
Male	38	43.2	38	43.2	76	43.2	
Female	50	56.8	50	56.8	100	56.8	
Race							0.833^a^
African American	9	10.2	12	13.6	21	11.9	
White	73	83.0	71	80.7	144	81.8	
Others	6	6.8	5	5.7	11	6.3	
Educational Attainment							0.002^a^*^^
Less than high school	1	1.1	1	1.1	2	1.1	
Some high school	1	1.1	1	1.1	2	1.1	
High school graduate	13	14.8	9	10.2	22	12.5	
Some college	9	10.2	14	15.9	23	13.1	
Four-year college graduate or higher	51	58.0	30	34.1	81	46.0	
No answer	13	14.8	33	37.5	46	26.1	
Employment Status							0.014^a^*^^
Employed	62	70.5	42	47.7	104	59.1	
Retired	10	11.4	13	14.8	23	13.1	
Disabled	1	1.1	2	2.3	3	1.7	
Unemployed	15	17.0	29	33.0	44	25.0	
No answer or unknown	0	0.0	2	2.3	2	1.1	
Marital Status							0.151^a^
Single	15	17.0	19	21.6	34	19.3	
Married	66	75.0	60	68.2	126	71.6	
Widowed	1	1.1	6	6.8	7	4.0	
Divorced/Separated	6	6.8	3	3.4	9	5.1	
Children <18 years old							0.010^b^*^^
No	57	64.8	73	83.0	130	73.9	
Yes	31	35.2	15	17.0	46	26.1	
Primary Insurance Status							0.215^b^
Public	17	19.3	25	28.4	42	23.9	
Private	71	80.7	63	71.6	134	76.1	
Housing Status							0.307^b^
Lives alone	6	6.8	11	12.5	17	9.7	
Lives with family or significant other	82	93.2	77	87.5	159	90.3	
Religious Affiliation							0.722^a^
Christianity	41	46.6	47	53.4	88	50.0	
Jewish	7	8.0	4	4.5	11	6.3	
Other (No answer or unknown)	15	17.0	13	14.8	28	15.9	
None	25	28.4	24	27.3	49	27.8	
Admission Type							.331^b^
Emergency	7	8.0	12	13.6	19	10.8	
Elective	81	92.0	76	86.4	157	189.2	
Hospital Service							.039^a^*^^
Neurosurgery, brain tumor	45	51.1	44	50.0	89	50.6	
Neurosurgery, spine	26	29.5	15	17.0	41	23.3	
Neurosurgery, vascular	12	13.6	15	17.0	27	15.3	
Orthopedic Surgery, spine	4	4.5	5	5.7	9	5.1	
Others	1	1.1	9	10.2	10	5.7	
Surgical Procedures							.060^a^
Craniectomy	5	5.7	11	12.5	16	9.1	
Craniotomy	28	31.8	24	27.3	52	29.5	
Microvascular decompression	7	8.0	2	2.3	9	5.1	
Decompression and fusion	3	3.4	3	3.4	6	3.4	
Deep brain stimulator placement	2	2.3	0	0.0	2	1.1	
Endoscopic resection of tumor	8	9.1	10	11.4	18	10.2	
Laminectomies, discectomies, and fusions	24	27.3	16	18.2	40	22.7	
Placement of epidural blood patch	2	2.3	1	1.1	3	1.7	
Ventriculoperitoneal shunt and revision	3	3.4	6	6.8	9	5.1	
Other surgical procedures	6	6.8	9	10.2	15	8.5	
Cranioplasty	0	0.0	6	6.8	6	3.4	
ESDP scores ≥10							.827^b^
No	75	85.2	77	87.5	152	86.4	
Yes	13	14.8	11	12.5	24	13.6	
Length of stay							.809^a^
1–7 days	81	92	82	93.2	163	92.6	
8–14 days	6	6.8	4	4.5	10	5.7	
≥15 days	1	1.1	2	2.3	3	1.7	

^a^ Fisher exact test; ^b^Pearson's chi-square test; statistical significance ^*^P < .05. *P* values in *bold* are statistically significant or significant.

ESDP, early screening for discharge planning.

The intervention group, who received face-to-face meeting interventions, had a TFU reach rate that was 21.6% higher than that of the comparison group. This indicated that pre hospital discharge face-to-face meeting interventions increased TFU reach rates, and Pearson's chi-square test demonstrated very high statistical significance (*P* = .000) (Table 2).

**Table 2.** Telephone Follow-Up Reach Rates and Phone Call Attempts

Call status	Intervention (n = 88)		Comparison (n = 88)		Total		
	n	%	n	%	N	%	*P*
Reach rate							**.000^a^**
Not reached	2	2.3	21	23.9	23	13.1	
Reached	86	97.7	67	76.1	153	86.9	
Total	88	100	88	100.0	176	100.0	
Number of phone call attempts							**.001^a^**
One	63	71.6	37	42	100	56.8	
Two	15	17	27	30.7	42	23.9	
Three	10	11.4	19	21.6	29	16.5	
Four	0	0	3	3.4	3	1.7	
Five	0	0	2	2.3	2	1.1	
Total	88	100	88	100.0	176	100.0	

^a^ Pearson's chi-square test. *P* values in *bold* are statistically significant.

The researchers also conducted a statistical analysis comparing the number of TFU attempts to successfully reach the patient. Close to 30% more patients on the intervention unit were reached during the first attempt compared to the comparison unit, which demonstrated statistical significance (*P* = .001) (Table 2). Interestingly, 4–5 call attempts were made to some patients (n = 5; 7.5%) on the comparison unit by other case managers trying to reach the patients successfully but this low number did not affect the results of this analysis (Table 2).

Table 3 describes the cross tabulation of subsequent ED visits of reached and not reached participants from the intervention and comparison groups. Participants in the intervention group who were reached successfully had 4.6% fewer subsequent ED visits than those in the comparison group who were successfully reached. The total ED visits of the reached participants were 7.8% (n = 12).

**Table 3.** Telephone Follow-Up Reach Rates, Emergency Department Visits, and Readmission Rates Between Groups

Call status	Intervention (n = 88)		Comparison (n = 88)		Total			
	n	%	n	%	N	%	*P*	
Reached							.290^b^; .368^a^; .450^c^	
With ED visit	5	5.8	7	10.4	12	7.8		
Without ED visit	81	94.2	60	89.6	141	92.2		
Not reached							.203^b^; .324^a^; .766^c^	
With ED visit	1	50.0	3	14.3	4	17.4		
Without ED visit	1	50.0	18	85.7	19	82.6		
Total ED visits							.294^b^; .292^a^;.432^c^	
With ED visit	6	6.8	10	11.4	16	9.1		
Without ED visit	82	93.2	78	88.6	160	90.9		
Reached							.276^b^; .255^a^; .527^c^	
Readmitted	4	4.7	1	1.5	5	3.3		
Not readmitted	82	95.3	66	98.5	148	96.7		
Not reached							.648^b^; 1.000^a^; 1.000^c^	
Readmitted	0	0.0	2	9.5	2	8.7		
Not readmitted	2	100.0	19	90.5	21	91.3		
Total Readmissions							.700^b^; .699^a^; 1.000^c^	
Readmitted	4	4.5	3	3.4	7	4.0		
Not readmitted	84	95.5	85	96.6	169	96.0		

^a^ Fisher exact test; ^b^Pearson's chi-square; ^c^Yates' continuity correction of the chi-square.

However, the ED visit rate for participants in the intervention group who were not successfully reached was close to 36% higher than that of the comparison group who were not reached. The total percentage of ED visits for participants not reached is 17.4% (n = 4), which is close to 10% higher in comparison to those patients who were successfully reached (Table 3).

Pearson's chi-square test also was also employed to determine whether successfully reaching adult surgical patients post hospital discharge would decrease the number of subsequent 30-day ED visits. The percentage of patients who were reached had lower rates of ED visits compared to those who were not reached, but the difference was not statistically significant (reached participants, *P* = .290; not reached participants, *P* = .203). Total ED visits also were analyzed and did not show a statistically significant difference between the groups (*P* = .294) (Table 3).

Because some cells contained 5 to 9 expected cases or counts and some cells had fewer than 5 expected count or cases, a Fisher exact test and Yates' continuity correction also were employed as a measure to ensure accuracy of results. For reached participants, Fisher exact test (*P* = .368) and Yates' continuity correction (*P* = .450) did not demonstrate any statistical significance. For not reached participants, Fisher exact test (*P* = .324) and Yates' continuity correction (*P* = .766) did not demonstrate any statistical significance. In addition, statistical significance was not demonstrated in total ED visits between the intervention and comparison group after employing a Fisher exact test (*P* = .292) and Yates' continuity correction (*P* = .432). Although not statistically significant, this finding is clinically significant and needs further investigation and research (Table 3).

## Discussion

This study is the first to examine TFU among neurosurgical patients. This also is the first study to employ inferential statistics to determine the effect of face-to-face meetings on telephone outreach for neurosurgical patients. The TFU reach rate for the intervention group was significantly greater (97.7%) than the reach rate for the comparison group (76.1%), with a difference of 21.6% and a very high statistical significance (*P* = .001). The 97.7% TFU reach rate was similar to that of a previous study that utilized face-to-face meetings before hospital discharge for medical patients.^6^ Past studies demonstrated TFU reach rates between 86% and 99% in medical patients.^6,17–20^ When compared to the average TFU reach rates (58%) of medical patients who did not receive face-to-face meeting interventions at the Johns Hopkins Hospital,^6^ the baseline reach rate for neurosurgical patients was still very high (76.1%) even though face-to-face meetings were not employed. Furthermore, the 76.1% TFU reach rate for the comparison group in this study was higher than the national average TFU reach rate (40%) in the United States^21^ and higher than the average TFU reach rate (68%) of a comparison group in a study conducted in an ED setting.^22^ This finding that, even without face-to-face meetings, TFU reach rates of neurosurgical patients were higher compared to TFU reach rates of medical and ED patients is clinically significant. The reasons for the underlying baseline difference of TFU reach rates between medical, ED, and neurosurgical patients are unknown, and warrant further research. Given that this is the first study of neurosurgical patients, replication of the study in a multisite setting and with different clinical specialties is highly recommended. Also, further investigation is needed to determine if sociodemographic variables and chronicity of illness between medical and neurosurgical patients are factors that affect patients being reached or not reached.

This study also explained that conducting pre hospital discharge face-to-face meetings may reduce the number of TFU attempts to successfully reach patients post hospital discharge. Many patients in the intervention group (n = 63; 71.6%) were reached during the first phone call attempt compared to the comparison group (n = 37; 42%), which demonstrated high statistical significance (*P* = .001). Most importantly, this is the first study to demonstrate that face-to-face meetings may reduce case managers' efforts to reach patients after hospital discharge.

The study findings demonstrated clinical significance but not statistical significance when examining ED visits and hospital readmissions rates, but the outcomes trended toward significance and are consistent with recent larger studies within the organization.^3,4,8^ These findings also were comparable to national benchmark clinical outcomes. Nationally, several studies and systematic reviews found that in the United States, readmission of neurosurgical patients with cranial procedures was between 6.9% and 23.89%.^23–26^ Average 30-day readmission rates following a neurosurgical spine procedure were between 4.2% and 7.4%.^27^

Readmissions of orthopedic spinal surgery patients varied by procedure but were generally lower than readmission rates of neurosurgical patients with cranial procedures and neurosurgical spine procedures. For example, readmission rates of patients who had orthopedic lumbar discectomies were 2.6%,^28^ and rates of patients who had lumbar fusions were around 2.9%.^29^ Nevertheless, the overall risks for neurosurgical and orthopedic complications and all-cause readmissions were similar across all types of procedures in a recently conducted retrospective longitudinal study.^30^

It also is important to understand the financial implication of TFU because of the current reimbursement trends and push for value-based care. Although the present study did not aim to calculate the specific financial impact of TFU on the neurosurgical ward, several studies within the researchers' organization demonstrated the value of TFU post hospital discharge as a part of a care coordination program.^2,3,4,6,8^ Overall, combining acute care and community interventions, the JCHiP program was associated with $113.3 million in cost savings between 2012–2016.^2^ Specifically, for the TFU service of the PAL department, 41,700 patients were served since 2013, covering 3 hospitals. There was a 29% relative reduction in readmissions from 2014–2017, which is equivalent to 777 readmissions prevented, totaling $11.8 million in cost savings.^2,31^ These cost reductions were achieved with 9 full-time PAL case managers who reached approximately 53% of eligible patients for TFU.^5^ There is room for improvement and huge potential for the TFU service to reduce costs exponentially if more patients are contacted, and patients who are hardest to reach and most likely to be readmitted are successfully reached.

### Limitations

The main limitation of this study was the lack of true randomization. This quasi-experimental study utilized a convenience sampling technique to obtain intervention group participants for face-to-face meetings. The design was unable to determine all preexisting factors that might have influenced the outcome of the results. For example, Early Screening for Discharge Planning Scores^32^ (whereby higher scores indicate potential need for special discharge services) were not considered before enrollment in the study because all patients, even those who declined the recommended high-intensity post-discharge services (eg, home care nurse visit, transition guide services) still received TFU from the telephonic nurse case manager.

Also, it was challenging to control the number of phone call attempts of other case managers conducting TFU on the comparison unit, leading to some patients being called more than 3 times. This study did not calculate the financial impact of TFU for neurosurgical patients. Furthermore, the sample in this study was obtained from 1 organization and 2 neurosurgical units in a large urban medical center, which limits generalization of results to other hospital settings and patient populations.

## Conclusion and Recommendations

This research study has laid the foundation for additional investigations into the impact of a face-to-face meeting on TFU reach rates, subsequent ED visits, and hospital readmissions for neurosurgical patients. This study increased TFU reach rates and decreased the frequency of phone call attempts by conducting face-to-face meetings with patients before hospital discharge. Although the results were not statistically significant in terms of decreasing subsequent ED visits and reducing hospital readmissions, the outcomes were clinically relevant, with an ultimate decrease in overall health care utilization. Further research is needed to fully understand the different factors associated with successfully reaching patients post hospital discharge. The significant and evolving roles of nurse case managers also warrant further investigation to better understand the impact of registered nurses on reducing health care utilization. There is also a need for discovery of innovative health care modalities that would increase patients' engagement in self-care management at home, which might lead to fewer subsequent ED visits and hospital readmissions, and result in reductions in health care utilization. Overall, it is essential to understand how to identify patients at highest risk for rehospitalization and to develop methods to engage the hardest to reach patients.
